# Combined Clavicular Hook Plate and Coracoid Screw Fixation for Coracoid Process Fractures Associated with Acromioclavicular Joint Dislocation

**DOI:** 10.3390/medicina62010212

**Published:** 2026-01-20

**Authors:** Bong Gun Lee, Young Seok Lee, Chang-Hun Lee, Wan-Sun Choi, Chang-Woo Woo, Young-Hoon Jo

**Affiliations:** 1 Department of Orthopaedic Surgery, Hanyang University College of Medicine, 222 Wangsimni-ro, Seoul 04763, Republic of Korea; 2 Department of Orthopaedic Surgery, Hanyang University Guri Hospital, 153 Gyeongchun-ro, Guri-si 11923, Republic of Korea; 3 Department of Orthopaedic Surgery, Ajou University School of Medicine, 164 World Cup-ro, Suwon 16499, Republic of Korea

**Keywords:** coracoid process fracture, acromioclavicular joint dislocation, clavicular hook plate, coracoid screw, outcomes

## Abstract

*Background and Objectives:* Coracoid process (CP) fractures combined with acromioclavicular (AC) joint dislocation are extremely rare, and evidence guiding optimal surgical management remains limited. This retrospective, single-center case series study evaluated clinical and radiologic outcomes after simultaneous fixation of both lesions using a clavicular hook plate and a coracoid screw. *Materials and Methods:* We retrospectively reviewed 15 consecutive patients with Ogawa type I CP fractures combined with AC joint dislocation who underwent clavicular hook plate and coracoid screw fixation between March 2019 and May 2024. Clinical outcomes at final follow-up included shoulder range of motion (ROM), visual analog scale (VAS) for pain, and the Constant score. Radiologic outcomes included CP union confirmed by computed tomography (CT) and residual AC joint subluxation. *Results:* The cohort comprised 13 men and 2 women with a mean age of 55.2 years, and the mean final follow-up was 40.2 months. At final follow-up, mean ROM was 168° for forward elevation, 161° for abduction, and 69° for external rotation at the side, with internal rotation to L1. The mean VAS score was 0.4 and the mean Constant score was 97. CT-confirmed union of the CP fracture was achieved in all patients, and no residual AC joint subluxation was observed. All patients returned to sports and activities of daily living. *Conclusions:* In this series, simultaneous fixation using a clavicular hook plate and a coracoid screw provided reliable stabilization for CP fractures with AC joint dislocation, achieving consistent CP union, restoration of AC joint alignment, and favorable clinical outcomes. However, given the retrospective, non-comparative study design, these findings should be interpreted with caution, and further comparative studies are warranted.

## 1. Introduction

Coracoid process (CP) fractures are relatively uncommon, accounting for approximately 3% to 13% of all scapular fractures, while scapular fractures represent about 1% of all fractures [1,2]. CP fractures are classified into two types according to their anatomic relationship with coracoclavicular (CC) ligament: Type I fractures occur proximal to the CC ligament, whereas Type II fractures occur distal to it [2]. Type I CP fractures are frequently associated with acromioclavicular (AC) joint dislocation, with a reported incidence of approximately 60% [2,3]. Type I CP fractures combined with AC joint dislocation often involve multiple disruptions of the superior shoulder suspensory complex (SSSC) as described by Goss [4], resulting in significant instability and frequently requiring surgical treatment [3,5].

However, CP fractures with AC joint dislocation are extremely rare, and current management relies on level IV and V evidence; therefore, standardized surgical treatment strategies have not yet been established [3,6]. Various surgical techniques have been described in the literature [7]. Some surgeons manage only the AC joint dislocation using Kirschner wires or a clavicular hook plate [8,9,10,11], while others perform fixation of the CP fracture alone with screws, aiming for indirect reduction in the AC joint [6,12]. In addition, several authors have reported simultaneous fixation of both the AC joint and CP fracture [13,14,15]. Clavicular hook plate combined with coracoid screw fixation has demonstrated favorable outcomes by stabilizing both lesions concurrently; however, most of the available evidence is limited to case reports [13,14,15]. Therefore, the clinical outcomes, radiologic outcomes, and complication rates associated with clavicular hook plate and coracoid screw fixation for CP fractures with AC joint dislocation remain unclear.

In this study, we retrospectively reviewed 15 patients with CP fractures combined with AC joint dislocation who underwent clavicular hook plate and coracoid screw fixation at our institution between March 2019 and May 2024. Clinical outcomes, radiologic outcomes, and complication rates were assessed to evaluate the effectiveness of this surgical approach.

## 2. Materials and Methods

### 2.1. Patients

This is a retrospective case series of patients with CP fractures combined with AC joint dislocation who underwent fixation with a clavicular hook plate combined with coracoid screw fixation. Because AC joint dislocation is generally not associated with Ogawa type II CP fractures, all patients included in this study had Ogawa type I injuries [3]. In contrast, CP fractures without AC joint dislocation were not a primary focus of this study, as management is typically directed toward the CP fracture itself and is relatively noncontroversial. Accordingly, we analyzed the effectiveness and outcomes of our surgical technique in this specific subset of injuries involving both CP fracture and AC joint dislocation.

Before adopting fixation with a clavicular hook plate combined with coracoid screw fixation, some patients with CP fractures accompanied by AC joint dislocation were treated using alternative techniques, including isolated screw fixation of the CP fracture and combined CP screw fixation with AC joint Kirschner wires stabilization. However, because the number of cases treated with these methods was limited and to maintain consistency of the surgical technique, patients treated with these alternative procedures were excluded from the present study.

Patients were identified through a retrospective review of the Hanyang University Guri Hospital registry between March 2019 and May 2024. The inclusion criteria were as follows: (1) acute CP fracture with AC joint dislocation (within 2 weeks of injury), (2) surgical treatment with a clavicular hook plate combined with coracoid screw fixation, (3) a minimum follow-up of at least 1 year, and (4) complete radiographic and clinical follow-up data available.

The exclusion criteria were as follows: (1) CP fractures without AC joint dislocation, (2) conservative management due to refusal of surgical treatment, (3) surgical treatment using techniques other than fixation with a clavicular hook plate combined with coracoid screw fixation, (4) concomitant injuries including clavicular fracture, acromion fracture, or other scapular fractures, (5) a history of shoulder disorders that could affect functional assessment, and (6) follow-up of less than 1 year.

### 2.2. Surgical Technique and Rehabilitation

**Figure 1:**
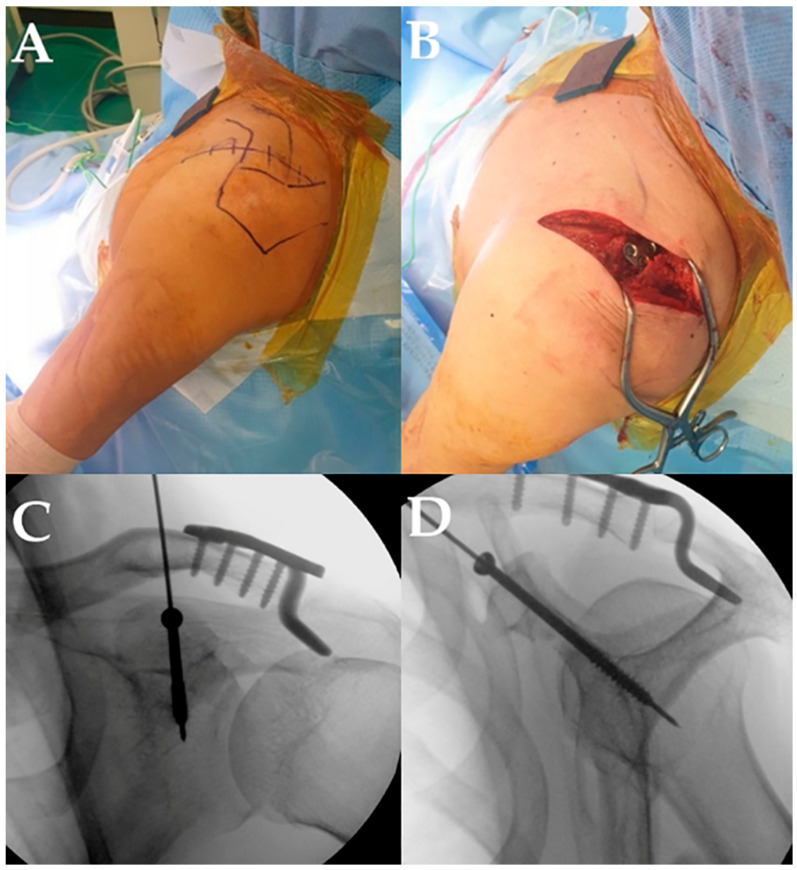
Surgical technique. (**A**) A longitudinal skin incision of approximately 10 cm was made along Langer’s lines, beginning approximately 2 cm proximal to the acromioclavicular (AC) joint and extending distally beyond the tip of the coracoid process (CP). (**B**) Through the proximal portion of the incision, the AC joint was exposed via the interval between the deltoid and trapezius muscles; through the distal portion, the CP was approached via the deltopectoral interval. (**C**,**D**) After guide pin insertion, the position was confirmed under C-arm fluoroscopy, and a 4.0 mm partially threaded cannulated screw was subsequently inserted.

The surgery was performed under general anesthesia, with the patient placed in the 70° beach chair position. A longitudinal skin incision of approximately 10 cm was made along Langer’s lines, beginning about 2 cm proximal to the AC joint and extending distally beyond the tip of the CP (Figure 1). We first reduced the AC joint using a clavicular hook plate (Jeil Medical, Seoul, Republic of Korea) prior to addressing the CP fracture. A skin flap was elevated bilaterally over the AC joint, and dissection was performed between the deltoid and trapezius muscles to expose the dislocated AC joint. The hook portion of the clavicular hook plate was inserted posterior to the AC joint, engaging the undersurface of the acromion. A 3.5 mm cortical screw was then inserted through the cortical hole of the plate to achieve reduction in the AC joint, with slight over-reduction being permitted. C-arm fluoroscopy was used to confirm adequate reduction in the AC joint, after which multiple locking head screws were inserted into the plate.

Subsequently, the deltopectoral approach was performed through the distal aspect of the incision, and the CP was exposed. Because type I CP fractures are located deep within the shoulder girdle and are surrounded by multiple ligamentous and tendinous attachments, direct visualization of the fracture site is technically challenging [6]. When the AC joint is reduced using a clavicular hook plate, the vertical displacement of the CP can be indirectly reduced. However, the deforming force of the conjoined tendon results in persistent flexion deformity of the CP; thus, extension of the CP facilitates reduction in the fracture site [6]. After fracture reduction, a guide pin was inserted 10 mm lateral to the medial margin of the CP and advanced along the scapular plane at an angle of 40° caudally and 10° medially [14,16]. Under C-arm fluoroscopic guidance, true anteroposterior and scapular Y views were obtained to confirm adequate fracture reduction and appropriate guide pin placement. Subsequently, a single 4.0 mm partially threaded cannulated screw (Synthes, West Chester, PA, USA) was inserted over the guide pin to achieve fixation of the CP.

The patients were immobilized in a Velpeau arm sling for 6 weeks postoperatively. Pendulum exercises were initiated on the first postoperative day. Passive range-of-motion (ROM) exercises were cautiously started 2 weeks after surgery. Active ROM exercises were initiated at 6 weeks postoperatively. While the clavicular hook plate was in situ, forward flexion and abduction beyond 90° were not recommended. In all patients, the clavicular hook plate was removed after radiographic confirmation of union of the CP fracture. Removal of the coracoid screw was performed only in patients who requested implant removal. Following implant removal, aggressive stretching exercises were encouraged to restore shoulder ROM. Once the shoulder ROM had recovered to a level comparable to that of the contralateral side, strengthening exercises were initiated.

### 2.3. Follow-Up and Evaluation

Follow-up evaluations were conducted at 2 weeks, 6 weeks, and 3 months postoperatively. Radiographic evaluation was performed using plain radiographs, including true anteroposterior (Grashey), scapular Y, and axillary views of the shoulder, in addition to anteroposterior and lordotic views of the clavicle. At 3 months postoperatively, computed tomography (CT) scans were obtained to assess fracture union, and union status was evaluated on axial and sagittal CT images. If fracture union was not confirmed at 3 months postoperatively, follow-up CT scans were performed at 2-month intervals until union was achieved. Once fracture union was confirmed on CT, implant removal was performed within 1 month of the confirmed union date.

After implant removal, patients were followed up at 2 weeks, 6 weeks, 3 months, 6 months, and 12 months, and annually thereafter. At each follow-up visit, radiologic evaluations were performed using plain radiographs only, and clinical outcomes were assessed using shoulder range of motion (ROM), the visual analog scale (VAS) score, and the Constant score. In addition, postoperative complications were recorded. Radiographic evaluation was used to assess union of the CP fracture and the presence of residual AC joint subluxation. Residual subluxation was defined as a vertical distance difference greater than 2 mm between the inferior margins of the acromion and the distal clavicle on the affected side compared with the contralateral side. This criterion was newly established in the present study because, in CP fractures accompanied by AC dislocation, the CC interval often remains within the normal range, and the CC interval may not adequately reflect residual AC joint subluxation.

## 3. Results

### 3.1. Patient Demographics and Injury Characteristics

A total of 15 patients (15 shoulders) who met the inclusion criteria were evaluated. There were 13 men and 2 women, with a mean age of 55.2 years (range, 34–67 years) (Table 1). The mean duration of final follow-up was 40.2 months (range, 12–66 months). The mechanisms of injury included falls in 8 patients, traffic accidents in 4 patients, and falls from height in 3 patients. The injured side was the right shoulder in 5 patients and the left shoulder in 10 patients. Surgery was performed at a mean of 4.7 days after injury (range, 0–9 days). All patients had Ogawa type I CP fractures combined with Rockwood type III AC joint dislocation. According to the Eyres classification, 12 patients had type III fractures, 1 patient had a type IV fracture, and 2 patients had type V fractures [17]. The clavicular hook plate was removed in all patients, whereas the coracoid screw was removed in 10 patients. The mean time to implant removal was 5 months postoperatively (range, 3.2–7 months).

### 3.2. Clinical Outcomes

At the final follow-up, the mean shoulder ROM was 168° for forward elevation (range, 150–180°), 161° for abduction (range, 140–170°), 69° for external rotation at the side (range, 50–80°), and internal rotation to the level of L1 (range, T7–L5). The mean visual analog scale (VAS) score was 0.4 (range, 0–2). The mean Constant score was 97 (range, 85–100). All patients achieved a full return to sports and activities of daily living. None of the patients experienced clinical symptoms of subcoracoid or subacromial impingement, AC joint deformity, or surgical site infection.

### 3.3. Radiologic Outcomes

**Figure 2:**
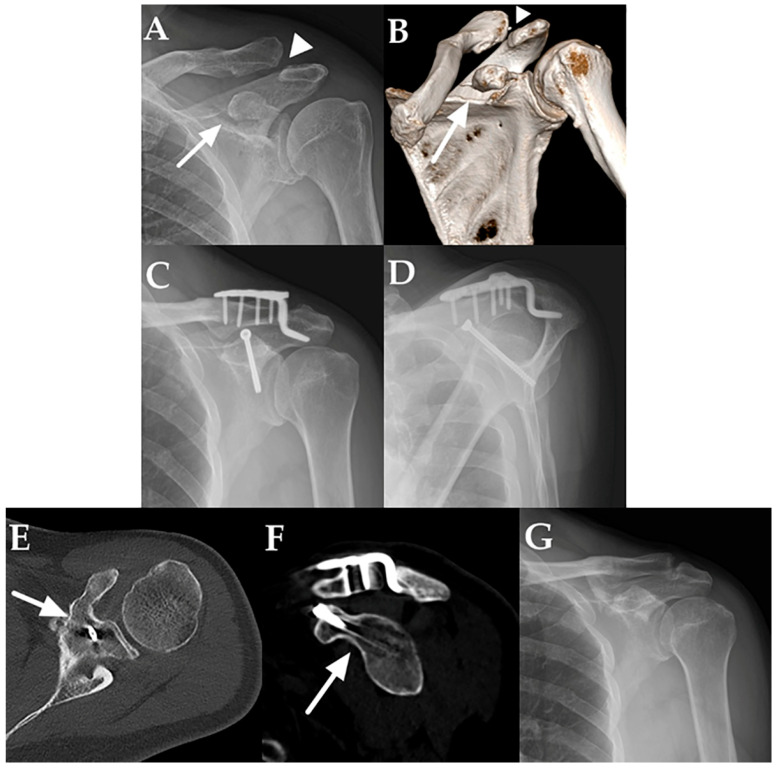
(**A**) Preoperative anteroposterior radiograph demonstrating a coracoid process (CP) fracture (arrow) with concomitant acromioclavicular (AC) joint dislocation (arrowhead). (**B**) Preoperative computed tomography (CT) with 3D reconstruction confirming the CP fracture (arrow) and associated AC joint dislocation (arrowhead). (**C**,**D**) Postoperative anteroposterior (**C**) and scapular Y view (**D**) radiographs demonstrating fixation of both lesions using a clavicular hook plate and a single 4.0 mm partially threaded cannulated screw. (**E**,**F**) CT images obtained 4.5 months postoperatively demonstrating complete union at the CP fracture site (arrow) on axial (**E**) and sagittal (**F**) views. (**G**) Follow-up radiograph after implant removal demonstrating no residual AC joint subluxation.

Union of the CP fracture was confirmed on axial and sagittal CT images in all 15 patients (Figure 2). The mean time to union was 4.6 months (range, 3–6.5 months). At the final follow-up, no residual AC joint subluxation was observed in any patient. Two patients had CP fractures involving the glenoid (Eyres type V); however, the articular step-off was ≤2 mm, and no radiographic evidence of post-traumatic arthritis was observed at the final follow-up.

### 3.4. Complications

A transient incomplete brachial plexus injury occurred in one patient postoperatively; however, complete spontaneous recovery was achieved within 3 months after surgery. Shoulder stiffness was observed in four patients at 3 months after implant removal; all patients subsequently recovered following ultrasound-guided glenohumeral joint steroid injection (triamcinolone, 40 mg) and stretching exercises. Subacromial erosion was observed in two patients, but no associated clinical symptoms were noted.

## 4. Discussion

CP fractures combined with AC joint dislocation are extremely rare injuries; therefore, standardized surgical treatment strategies have not yet been established [3,7]. Consequently, various surgical approaches have been reported in the literature [7]. In the present study, we treated 15 patients with CP fractures combined with AC joint dislocation by stabilizing the AC joint using a clavicular hook plate and fixing the CP fracture with a single 4.0 mm partially threaded cannulated screw. Fracture union of the CP was achieved in all patients, with no residual AC joint deformity observed. Overall, clinical outcomes were favorable in all cases.

The optimal treatment strategy for CP fractures combined with AC joint dislocation remains controversial, as some authors advocate surgical intervention, whereas others prefer conservative management [1,5,18]. The SSSC comprises a ring of bony and soft-tissue structures, including the acromion, AC joint, distal clavicle, CC ligaments, CP, and glenoid [4]. These structures play an important biomechanical role in maintaining a stable connection between the upper extremity and the axial skeleton [19]. An isolated injury to the SSSC does not significantly compromise the stability of the ring; however, disruption at two or more sites compromises its structural integrity and may cause complications such as delayed union, non-union, loss of strength, and degenerative arthritis [4,9,20]. CP fractures combined with AC joint dislocation represent unstable injuries involving double disruption of the SSSC. Conservative treatment in these combined injuries has been reported to be associated with CP non-union, persistent AC joint pain, and cosmetic deformity [3,21]. Therefore, surgical treatment is generally required, except in cases with minimal displacement of the CP fracture [5,11,13].

Several authors have reported surgical strategies in which only one of the disrupted lesions is treated in CP fractures combined with AC joint dislocation [8,9,12]. By converting a double disruption of the SSSC into an isolated injury through surgical stabilization of a single lesion, the stability of the ring can be restored. Accordingly, some surgeons have focused on stabilization of the AC joint alone [8,9,10,11], whereas others have described fixation of the CP fracture alone [6,12]. Surgical stabilization of the AC joint alone is technically less demanding and carries a lower risk of neurovascular injury than isolated fixation of the CP fracture [8,9,10]. Moreover, reduction in the AC joint may facilitate indirect reduction in the CP fracture, and satisfactory clinical outcomes following this approach have been reported [8,9,10]. However, even after reduction in the AC joint, the CP fracture may remain unstable and develop a flexion deformity due to persistent deforming forces of the conjoined tendon, potentially leading to non-union [6,8,11]. Consistent with this concern, previous studies have reported CP non-union rates ranging from approximately 16.7% to 22% after isolated stabilization of the AC joint, along with additional complications such as residual AC joint subluxation and deformity [7,8].

Although satisfactory clinical outcomes have been reported even in cases of CP non-union [8,11], previous studies have described patients with type I CP non-union who experience significant pain [21,22]. In particular, unstable type I CP non-union may cause deformity of the coracoacromial arch, potentially leading to subacromial impingement [21]. In addition, CP non-union involving the glenoid has been associated with the development of glenohumeral arthritis and may result in shoulder stiffness accompanied by nocturnal pain [21]. Because revision surgery for CP non-union is technically more complex [7], surgeons should make every effort to achieve fracture union of the CP during the initial surgical procedure.

In our experience, reduction in the AC joint using a clavicular hook plate resulted in indirect reduction in the vertical displacement of the CP fracture; however, residual flexion deformity and fracture instability frequently persisted [8]. Accordingly, the CP was extended to correct the flexion deformity, followed by fixation of the fracture with a single cannulated screw [6]. Because the clavicular hook plate reduces the tensile force of the CC ligament on the CP, sufficient fixation strength of the CP fracture can be achieved using a single cannulated screw [15]. Simultaneous fixation of both lesions provides rigid stabilization and has been associated with high union rates of CP fractures in the literature [13,14,15,23]. Consistent with these reports, fracture union was achieved in all 15 cases in our series, with favorable clinical outcomes.

In CP fractures combined with AC joint dislocation, isolated fixation of the CP fracture is a feasible surgical option [6,7,12]. Stabilization of the CP fracture may allow indirect reduction in the AC joint through the intact CC ligaments [12]. Furthermore, avoidance of AC joint fixation may decrease the incidence of AC joint–related complications, including subacromial erosion, while obviating the need for a secondary procedure for implant removal [24]. However, isolated fixation of the CP fracture is technically demanding [9,10]. Type I CP fractures are located deep within the shoulder girdle and are surrounded by multiple ligamentous and tendinous attachments, making direct visualization of the fracture site difficult [6,25]. Consequently, achieving and maintaining anatomic reduction while simultaneously inserting fixation screws can be challenging.

In contrast, initial stabilization of the AC joint using a clavicular hook plate enables indirect reduction in the vertical displacement of the CP fracture [8,9]. Once vertical alignment has been restored, only correction of the residual flexion deformity of the CP is required, which facilitates fracture reduction and simplifies screw fixation. Therefore, this stepwise approach makes the procedure technically easier, enabling the surgeon to focus primarily on accurate screw placement. Moreover, the use of a clavicular hook plate contributes to restoration of horizontal stability of the AC joint in the setting of AC joint dislocation [13]. Simultaneous fixation of both lesions may help restore the integrity of the SSSC ring, maintain reduction, and reduce secondary deformities such as residual AC joint subluxation [13,15,23]. Nevertheless, further biomechanical investigations and larger comparative studies are warranted to confirm the benefits of this surgical strategy.

In our study, several postoperative complications were observed. One notable complication was a transient incomplete brachial plexus injury. Although the exact mechanism could not be definitively determined, this complication was presumed to be associated with excessive medial retraction during exposure of the CP for fixation of the CP fracture, as well as with factors related to intraoperative patient positioning [26,27]. Fortunately, the neurologic deficit resolved completely within 3 months postoperatively. Meticulous attention should be paid to avoiding excessive retraction at the surgical site, as well as to proper patient positioning to prevent excessive cervical stretching during the procedure.

Other postoperative complications included subacromial erosion and shoulder stiffness. These complications were associated with the use of the clavicular hook plate [24,28]. In the present study, the incidence of subacromial erosion was lower than that reported in previous studies [8,29], which may be attributable to the use of clavicular hook plates bent approximately 15°, thereby reducing subacromial contact pressure [30]. Importantly, subacromial erosion did not adversely affect the final clinical outcomes [29]. In addition, all cases of shoulder stiffness resolved with conservative management following implant removal.

This study has several strengths. CP fractures combined with AC joint dislocation are extremely rare injuries, and this study reports a relatively large series of patients treated using a single, standardized surgical strategy. To the best of our knowledge, this is the largest case series to date in which both lesions were simultaneously stabilized. In addition, fracture union was confirmed using CT in all cases, providing objective radiologic evidence of successful CP union.

Nevertheless, this study has several limitations. First, the retrospective design is inherently susceptible to selection bias. Second, the relatively small sample size—reflecting the rarity of this injury pattern—limited meaningful comparisons with alternative surgical strategies, such as isolated fixation of either the AC joint or the CP fracture. Third, the lack of a control group limits our ability to draw definitive conclusions regarding the superiority of the described technique. Fourth, although the Constant score is widely used, a potential ceiling effect should be acknowledged, as many patients achieved near-maximum scores in our cohort [31]. Finally, we defined residual AC joint subluxation as a ≥ 2 mm difference in the distance between the inferior margins of the distal clavicle and the acromion compared with the contralateral side. However, this cutoff was arbitrarily chosen to account for potential measurement error and should not be considered an absolute criterion, as some variability is possible.

## 5. Conclusions

In this retrospective case series, clavicular hook plate fixation combined with coracoid screw fixation was associated with consistent fracture union, restoration of AC joint alignment, and favorable clinical outcomes in patients with CP fractures accompanied by AC joint dislocation. Simultaneous fixation of both lesions may provide rigid stabilization and may help reduce CP non-union and residual AC joint deformity in this rare and unstable injury pattern. However, given the non-comparative design and the need for routine hook plate removal, these findings should be interpreted cautiously. Prospective comparative studies are warranted to define the relative effectiveness of this strategy compared with alternative techniques.

## Figures and Tables

**Table 1:** Patient demographics, injury characteristics, and clinical and radiologic outcomes.

No.	Sex	Age (y)	Follow-Up (mo)	Mechanism	Side	Eyres Type	Removal Time (mo)	VAS Score	Constant Score	CP Union	Union Time (mo)	Complications
1	M	55	30	FH	Right	III	4.7	1	85	Yes	4.5	
2	M	66	66	Fall	Left	III	5	2	89	Yes	4.6	BPI
3	M	50	54	TA	Right	III	6.2	0	100	Yes	5.4	Stiffness
4	F	49	52	Fall	Left	III	4.8	0	100	Yes	4.7	
5	M	45	50	TA	Left	III	4.7	0	96	Yes	4.5	Stiffness
6	M	67	48	TA	Left	III	4.7	0	98	Yes	4.5	Stiffness
7	M	63	43	Fall	Left	V	7	0	100	Yes	6.5	
8	M	40	51	TA	Left	IV	3.2	0	96	Yes	3.1	
9	M	63	50	Fall	Left	III	3.2	2	95	Yes	3	Stiffness
10	M	54	30	FH	Right	III	5.5	0	100	Yes	5.2	SE
11	M	61	42	Fall	Left	III	5	0	100	Yes	4.6	
12	F	55	40	Fall	Right	III	5.2	0	100	Yes	4.7	
13	M	59	17	Fall	Left	III	4.7	1	95	Yes	4.5	SE
14	M	34	18	FH	Right	III	5.5	0	100	Yes	4.7	
15	M	67	12	Fall	Left	V	5.5	0	100	Yes	4.6	

M, male; F, Female; FH, fall from height; TA, traffic accident; CP, coracoid process; BPI, brachial plexus injury; SE, subacromial erosion.

## Data Availability

The datasets used and/or analyzed in the current study are available from the corresponding author upon reasonable request.
